# Paediatric facial fractures

**DOI:** 10.1007/s40368-025-01134-2

**Published:** 2025-10-29

**Authors:** Ella Starck, Esa Färkkilä, Eeva Kormi, Juho Suojanen

**Affiliations:** 1 Faculty of Medicine, Clinicum, University of Helsinki, Helsinki, Finland; 2 Department of Oral and Maxillofacial Surgery, Päijät-Häme Joint Authority for Health and Wellbeing, Päijät-Häme Central Hospital, Lahti, Finland; 3 Department of Plastic Surgery, Cleft Palate and Craniofacial Centre, Helsinki University Hospital, Helsinki, Finland

**Keywords:** Facial fracture, Facial trauma, Paediatric trauma, Trauma mechanism, Children trauma

## Abstract

**Background:**

Paediatric facial fractures are relatively rare due to the elasticity of children´s bones. This elasticity can also make such fractures more challenging to diagnose. Additionally, anatomical differences between juveniles and adults influence the types of fracture observed. In general, boys are more susceptible to trauma; however, this trend does not seem to differ in cases of facial fractures.

**Purpose:**

The aim of this study is to investigate the association between sex and trauma mechanisms in paediatric facial fractures.

**Methods:**

A retrospective cohort study was conducted on paediatric patients diagnosed with facial bone fractures between 2008 and 2018 at Päijät-Häme Central Hospital (Lahti, Finland). Inclusion criteria were one or more fractures in facial bones in patients under 18 years of age.

**Results:**

Of the 37 study subjects, 19% were female and 81% were male. Among females, the most common trauma mechanisms were bicycle accidents and sports related incidents (both 8.1%), whereas in males, motor vehicle accidents and sports related incidents were equally prevalent (both 24%). Associated injuries were uncommon, occurring only in 16% of cases, and they were particularly linked to motor vehicle accidents.

**Conclusion:**

Paediatric facial fractures are rare. Mandibular fractures are more frequent in both the 6–12 and 13–18-year age groups. No consistent association between dentition stage and fracture site is observed. In both sexes, sports related incidents are the most common trauma mechanism, followed by bicycle accidents in girls and MVAs in boys. Differences between trauma mechanisms and facial fracture locations are minor. Associated injuries are infrequent and typically occur in connection with high-energy trauma, such as MVAs.

## Introduction

Facial fractures are less common in paediatric patients than adults due to their more elastic bone and cartilage structure (Rogan et al. 2024; Rogan and Fang 2024; Vyas et al. 2008). However, when occurring, facial fractures often tend to be more severe and can result in life-long consequences such as disability or even death (Braun et al. 2017; Totonchi et al. 2012). As in adults, paediatric patients with facial fractures are more often male than female (Kaura et al. 2018; Rogan et al. 2024; Vyas et al. 2008).

Fracture patterns in paediatric patients are age-related and differ from older patients. The most significant factors contributing to this are expanding sinuses and erupting teeth which are characteristic of certain age groups. (Rogan et al. 2024; Totonchi et al. 2012) Incomplete pneumatization signifies thicker bone structure and incomplete dentition strengthens the jawbones. Thus, both stabilize facial structures. (Rogan et al. 2024) In children, oblique fractures are the most common fracture pattern, whereas adults more often present with more horizontal Le Fort fractures (Naran et al. 2016). Facial fractures can appear in the upper face (frontal bone), the midface (nasal bone, orbit, maxilla, zygoma) and the lower face (mandible, alveolar ridge, teeth).

Younger children have a proportionally larger skull than face which is why it is more common to encounter trauma in the cranial part than the midface (Imahara et al. 2008; Rogan and Fang 2024; Vyas et al. 2008). In older paediatric patients, fractures in the mandible are more likely (Imahara et al. 2008). Due to the elastic cartilage and more pliable structure in immature bones, children’s fractures tend to be less displaced than in adult patients and may often appear as greenstick fractures (Chasm and Swencki 2010). Another characteristic appearance in children is a greenstick type blowout fracture, in which orbital contents, usually the inferior rectus or the inferior oblique muscle, become trapped between broken orbital bones when they snap back into their place after breaking (Phan et al. 2012). This type of fracture is called trapdoor, and it is due to elasticity of orbital bones (Egbert et al. 2000).

Trauma in paediatric patients is most often caused by a blunt force, though penetrating injuries are also possible (Rogan and Fang 2024). Blunt trauma can originate from falls, traffic or sports accidents, and assaults for example. Several studies have shown traffic accidents to be the most common etiological factor (Imahara et al. 2008; Kirvelä et al. 2024). In younger children, assaults often refer to child abuse. Regarding trauma aetiology, accidental self-injurious behaviour in younger paediatric patients, and suicide attempts in older patients should be kept in mind (Rogan and Fang 2024). Fracture prevalence grows with age and is most common in adolescents aged 12–18 years (Braun et al. 2017; Grunwaldt et al. 2011; Imahara et al. 2008). The fact that children have several factors protecting them from facial fractures denotes that a major force is often associated when occurring. Therefore, it is important to pay attention to concomitant injuries when examining facial fractures, especially noticing the airways, brain, eyes and neck.

When suspecting a facial fracture, computed tomography (CT) is commonly used to confirm the diagnosis (Rogan and Fang 2024). What comes to treatment, it is essential to consider the ongoing growth and development of paediatric patients. This is why paediatric fractures are often treated conservatively, even if surgical treatment for similar fractures in adults would be required (Braun et al. 2017). However, when mandatory to operate, the growth centres should be preserved untouched (Singh and Bartlett 2004). In addition, resorbable plates and screws should be used when treating a fracture operatively (Burlini et al. 2015). Due to continuing growth, children with facial fractures should be followed for a longer time since it may affect growth, and intervention with orthodontics may be needed (Braun et al. 2017; Naran et al. 2016; Wheeler and Phillips 2011). Overall, the prognosis for facial fractures in paediatric patients is mostly good due to the outstanding capability of remodelling. As a result, lasting bone harm or surgical approach is infrequent. (Rogan and Fang 2024).

Knowledge of how injury patterns affect paediatric maxillofacial fracture patterns is limited. Additional information about this might help reducing and preventing risks for facial fractures. This is a descriptive study of paediatric patients and their characteristic appearances in facial fractures, treatment and coefficient factors. The aim is to investigate if sex associates with trauma mechanisms and if certain trauma mechanisms lead to specific facial fractures. The hypothesis is that sex has an influence on trauma mechanisms and that trauma mechanisms and facial fractures have a connecting pattern.

## Materials and methods

A retrospective cohort study of paediatric patients with facial fractures treated at Päijät-Häme Central Hospital (PHCH, Lahti, Finland) was conducted. Data were collected for a 10-year period, from 2008 to 2018. The study was approved by the institutional review board of PHCH (D/18/07.01.04.05/2018 and D/2929/07.01.04.05/2020). Patients were identified from PHCH’s patient register using the International Classification of Diseases, Ninth Revision (2007–2014) codes 802, 805.0–805.18, 806.0–806.19 and 847.0 and Tenth Revision (2015–2017) codes S02.2-S02.04 and S02.6-S02.7, which correspond to fractures in the upper face, midface, and mandible. Paediatric patients were defined as individuals under 18 years old. The data were collected using Microsoft Excel, and the figure was created using Microsoft OneNote and Microsoft PowerPoint.

The inclusion criteria were one or more fractures in facial bones in minors (< 18 years of age) with complete medical and imaging records who were treated at PHCH. Fractures alone in the alveolar ridge were excluded, as well as patients whose later examination ruled out the diagnosis in demand, referring to initially misdiagnosed fractures. The cohort contained patient’s age, sex, examination date, fracture location(s), mechanism of injury, associated injuries (AIs), Glasgow Coma Scale (GCS), and treatment. Fractures in the upper face included frontal fractures, while midfacial fractures included orbital, maxillary, nasal and zygomatic fractures, and lower facial fractures consisted of mandibular fractures. Mandibular fracture sites were further categorized based on radiographic imaging as follows: ramus-condyle unit (RCU), coronoid, angle, body and symphyseal/parasymphyseal region. The dentition status was categorised into primary dentition, early mixed dentition, late mixed dentition, and permanent dentition. Trauma mechanisms were divided into motor vehicle accidents (MVA), bicycle accidents, sports related incidents, assaults and falls. AIs were classified into extremity injuries, intracranial haemorrhages, skull fractures, thoracic injuries, blunt cerebrovascular injuries, cervical spine injuries, diffuse axonal injuries or concussions, and pelvic or lumbar spine injuries.

## Results

During the study period, 481 patients with facial fractures, complete medical records and imaging data were found in the patient register. Of these, 37 subjects were paediatric and met the inclusion criteria, forming our study cohort.

Of these subjects, 7 (19%) were female and 30 were male (81%), leading to a male to female ratio of 4.3:1. Subjects’ ages were from 6.0 to 17.9 years, with a mean of 14.3 years. Females´ ages ranged from 6.0 to 16.1 (mean of 12.3 years), whereas males’ ages ranged from 8.0 to 17.9 years (mean 14.7 years). The median age of all patients was 15.4 years; 12.4 years in females and 15.5 years in males. Demographic data are presented in Table 1.

**Table 1:** Demographic data

	*N* (%)	Range (years)	Mean (years)	Median (years)
All	37 (100)	6.0–17.9	14.3	15.4
Female	7 (19)	6.0–16.1	12.3	12.4
Male	30 (81)	8.0–17.9	14.7	15.5

There were 22 subjects with mandibular fractures (60%). Of these, 12 (32%) had a single fracture, 6 (16%) had at least one additional fracture in the mandible, and 4 (11%) had a bilateral fracture in the RCU. Of all subjects, 15 (41%) had fractures in the RCU, 8 (22%) in the symphysis or parasymphysis, 6 (16%) in the angle, and 1 subject (2.7%) had a fracture in the body of the mandible. Of combined fractures, 3 subjects (8.1%) had fractures in the alveolar ridge.

**Fig. 1:**
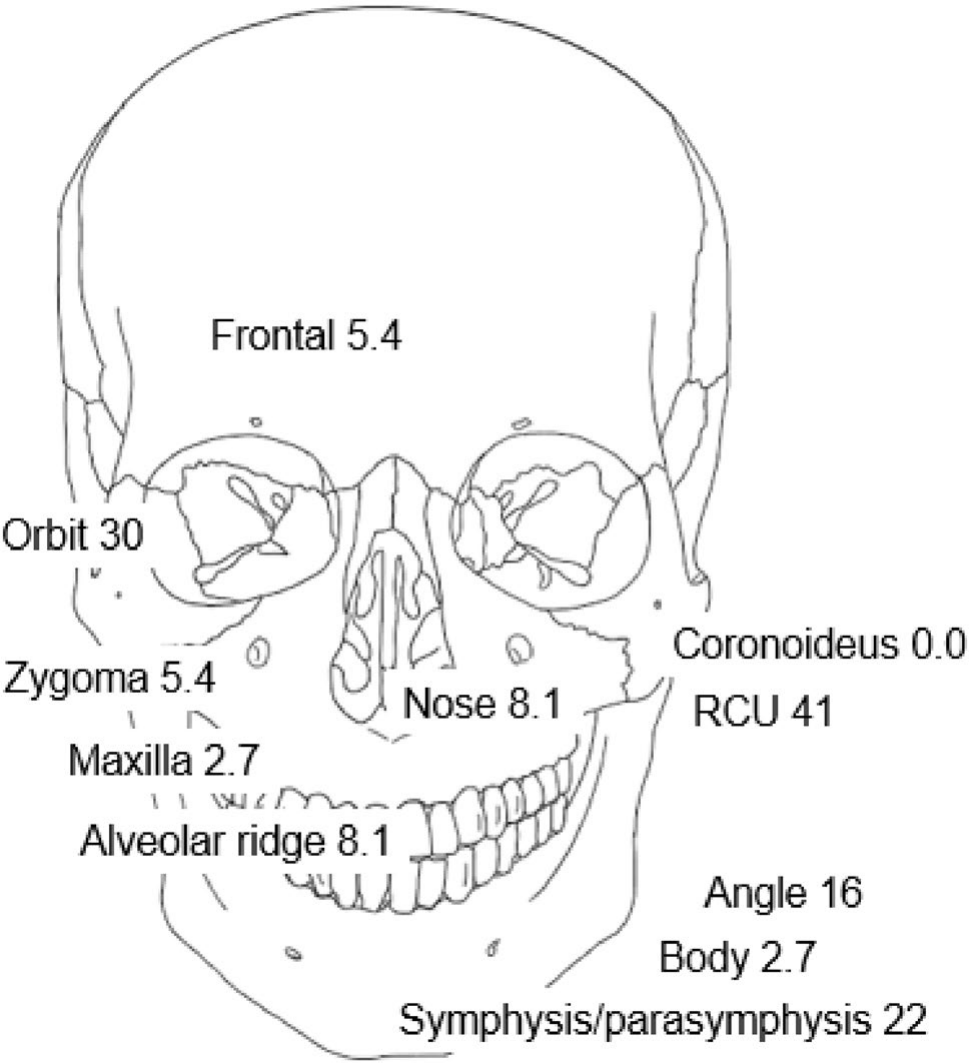
Prevalence of different paediatric facial fractures (%), the most common fracture site was the ramus-condyle unit (RCU) in the mandible, followed by the midfacial bony orbit and the mandibular symphysis/parasymphysis area. All alveolar fractures were combined with other craniomaxillofacial fractures

In total, 15 subjects (41%) had fractures in the midface or upper face. There were 13 subjects (35%) with midfacial fractures of which 10 (27%) had isolated fractures and the rest 3 of them (8.1%) combined midfacial fractures. Midfacial fractures appeared in the orbit in 11 subjects (30%), in the nose in 3 (8.1%), in the zygoma in 2 (5.4%), and in the maxilla in 1 subject (2.7%). Only 2 subjects (5.4%) encountered fractures in the upper face, both of them in the frontal bone. Fracture locations and distributions are illustrated in Fig. 1. Altogether, 25 (68%) of the facial fractures were unilateral and 12 (32%) bilateral. Of the unilateral fractures, 20 (54%) occurred on the left side and 5 (14%) on the right side of the head.

Regarding dentition status, 3 subjects (8.1%) had primary dentition, 6 (16%) had early mixed dentition, 1 (2.7%) had late mixed dentition, and 27 subjects (73%) had permanent dentition. Among subjects with primary dentition, 2 had mandibular fractures and 1 had a midfacial fracture. In subjects with early mixed dentition, fractures occurred 4 times in the mandible and twice in the midface. The subject with late mixed dentition had a mandibular fracture. Of the 27 subjects with permanent dentition, 15 had mandibular fractures and 12 had fractures in the midface or upper face.

Sports related incidents caused facial fractures in 12 subjects (32%), MVAs in 10 (27%), bicycle accidents in 9 (24%), assaults in 4 (11%) and falls in 2 of the subjects (5.4%). Table 2 presents the division of different trauma mechanisms, fracture types, sex, and AIs. MVAs (14%) and falls (2.7%) caused an equal number of fractures in the midface or upper face as in the mandible, whereas all assaults (11%) and most bicycle accidents (19%) resulted in mandibular fractures. Of 12 sports incidents, 7 (19%) led to midfacial or upper facial fractures and 5 (14%) to mandibular fractures.

**Table 2:** Trauma mechanism distribution by fracture type, sex and AIs

	Total (%)	Midface/ Upper face, *n* = 15 (41%)	Mandible, *n* = 22 (60%)	Female *n* = 7 (19%)	Male *n* = 30 (81%)	AI, *n* = 6 (16%)
Sport	12 (32)	7 (19%)	5 (14%)	3 (8.1%)	9 (24%)	2 (5.4%)
MVA	10 (27)	5 (14)	5 (14)	1 (2.7)	9 (24)	4 (11)
Bicycle	9 (24)	2 (5.4)	7 (19)	3 (8.1)	6 (16)	0 (0.0)
Assault	4 (11)	0 (0.0)	4 (11)	0 (0.0)	4 (11)	0 (0.0)
Fall	2 (5.4)	1 (2.7)	1 (2.7)	0 (0.0)	2 (5.4)	0 (0.0)

*MVA* motor vehicle accident, *AI* associated injury

Among females, the most common trauma mechanisms were bicycle accidents together with sports related incidents (both 8.1%), followed by one (2.7%) MVA-related fracture. Among males, the most common mechanisms were MVAs together with sports incidents (both 24%), followed by bicycle accidents (16%), assaults (11%) and falls (5.4%).

AIs were present in 6 subjects (16%). Of these, 4 (11%) appeared in subjects with midfacial fractures and 2 (5.4%) in subjects with mandibular fractures. AIs were caused by MVAs in 4 subjects (11%) and sports incidents in 2 subjects (5.4%). AIs included extremity trauma in 4 subjects (11%), and intracranial haemorrhage (ICH), skull fracture, and thoracic injury in 2 subjects each (5.4%). None of our subjects sustained blunt cerebrovascular injuries, cervical spine injuries, diffuse axonal injuries, concussions, or pelvic or lumbar spine injuries.

When subjects were divided into school-aged children (6–12 years) and adolescents (13–18 years), the prevalence was clearly higher in the latter group, with 28 subjects (76%). In contrast, two-thirds of the fractures in the younger age group occurred in the mandible, whereas in the older age group, the distribution between mandibular fractures and fractures in the midface or upper face was more balanced, mandibular fractures being slightly more common. Regarding trauma mechanisms, bicycle accidents were clearly the most frequent cause of fractures in school-aged children, while sports incidents and MVAs were the leading trauma mechanisms among adolescents (Table 3).

**Table 3:** Age distribution by fracture type and trauma mechanism

	Midface/ Upper face, *n* = 15 (41%)	Mandible, *n* = 22 (60%)	Sport (%)	MVA (%)	Bicycle (%)	Assault (%)	Fall (%)
6–12 years *n* = 9 (24%)	3 (8.1%)	6 (16%)	1 (2.7)	2 (5.4)	6 (16)	0 (0.0)	0 (0.0)
13–18 years *n* = 28 (76%)	12 (32)	16 (43)	11 (30)	8 (22)	3 (8.1)	4 (11)	2 (5.4)

*MVA* motor vehicle accident

Radiographic examination was most often performed using either CT (53%) or panoramic radiograph (45%), and both in one subject (2.7%). All midfacial fractures were diagnosed using CT as the imaging modality. Panoramic radiography was used to diagnose mandibular fractures. During the study period, no significant changes were identified in the imaging modalities used.

Operative treatment was performed in 19 subjects (51%). Of these, 13 (35%) had mandibular fractures, including 7 (19%) with condylar fractures—5 (14%) unilateral and 2 (5.4%) bilateral. The remaining operated mandibular fractures were located in the dental region of the mandible. Among patients with midfacial fractures, operative treatment was chosen in 6 cases (16%): 1 (5.4%) with a naso-orbito-ethmoid (NOE) fracture, 1 (5.4%) with a zygomatic fracture, 1 (5.4%) with combined maxillary and nasal fractures, and 3 subjects (8.1%) with orbital blowout fractures, including 1 (5.4%) trapdoor fracture.

All subjects had a normal GCS of 15. With regard to injury timing, 5 (14%) occurred in winter, 11 (30%) in spring, 13 (35%) in summer and 8 (22%) in autumn.

## Discussion

The aim of this study was to investigate whether sex is associated with trauma mechanisms and if certain trauma mechanisms lead to specific facial fractures in paediatric patients. The hypothesis was that sex has an influence on trauma mechanisms and that trauma mechanisms and facial fractures have a connecting pattern. We also examined the presence of characteristic patterns in facial fractures, treatment, and contributing factors.

To begin with, the difference in incidence between female and male subjects was remarkable, as there were approximately four times more boys than girls. This was expected, since males are generally more likely to experience accidents and facial trauma, which has been stated in various other studies as well (Goswami 2024; Kaura et al. 2018; Khan et al. 2019; Vyas et al. 2008; Wusiman et al. 2020). Goswami’s study reported a male-to-female ratio in children aged 12 years or less of 1.6:1 (Goswami 2024). The difference compared to our 4.3:1 ratio could be explained by the fact that older boys (> 12 years) are more often involved in contact sports or other higher-risk free time activities, such as motocross, than girls.

This also explains the slightly higher mean age in males compared to females. Differing from some other studies of facial fractures in paediatric patients, our subjects were all 6 years or older. This is because facial fractures are very rare in the youngest age group due to their flexible bone and cartilage structure, the relatively dominant skull size, and the protective frontal bone (Alhumsi and Gilardino 2014; Rogan et al. 2024; Rogan and Fang 2024; Vyas et al. 2008). Moreover, the age distribution was clearly more emphasized in adolescents than in younger children, which aligns with previous studies (Ferreira et al. 2005; Grunwaldt et al. 2011). This is due to progressive independence and greater involvement in contact sports and driving, as seen in our study, but also supported in other studies (Braun et al. 2017; Grunwaldt et al. 2011). In contrast, bicycle accidents were the most common cause of facial fractures in school-aged children, which is consistent with the fact that ages 6 to 12 are typically when children learn to ride a bicycle.

Over half (60%) of the subjects’ facial fractures emerged in the mandible, making it clearly the most common fracture site. This is in line with other studies about maxillofacial fractures in both paediatric patients and adults (Goswami 2024; Khan et al. 2019; Wusiman et al. 2020). The mandible is prone to fractures due to its prominent appearance and low facial position (Wusiman et al. 2020). We found that fractures in the RCU were the most common (41%), followed by fractures in the symphysis or parasymphysis area (22%). This is consistent with studies of paediatric mandibular fractures by Smith et al., who found RCU fractures in 56% and symphysis or parasymphysis fractures in 27% of the subjects, and by Steed et al., who also stated that mandibular fractures occur most often in the RCU, followed by symphysis or parasymphysis (Smith et al. 2013; Steed and Schadel 2017). When comparing our study to those involving the whole population, Gualtieri et al.’s mandibular fracture sites aligned with ours, whereas Wusiman et al. found the symphysis or parasymphysis to be the most commonly fractured in adults, though followed with condyle fractures (Gualtieri et al. 2021; Wusiman et al. 2020).

The remaining facial fractures were mostly midfacial, with orbital fractures being distinctly the most common (30%) due to the orbit´s fragile, paper-thin bone structure (Felding 2018). However, only one of our subjects was diagnosed with an orbital trapdoor fracture (2.7%), although other studies report an incidence between 24 to 40% in paediatric patients (Bansagi and Meyer 2000; Chi et al. 2010). The low percentage could be because trapdoor fractures are acute conditions that require immediate treatment and are therefore directed forward from PHCH if there is not a maxillofacial surgeon on call at that instant. The nasal bone, which is often fractured in adults, was fractured in only 8.1% of subjects. This aligns with Landeen et al. who stated that nasal fractures are less common in paediatric patients than in adults (Landeen et al. 2022). The frontal bone was fractured in 2 subjects (5.4%), which corresponds with other studies reporting a frequency of 5–15% for frontal fractures (Marinheiro et al. 2014; Schultz et al. 2017).

In contrast to our study, where none of the patients it the younger age group had an upper facial fracture, earlier studies have reported that in younger patients the cranial part is more frequent to fracture than the midface (Imahara et al. 2008; Rogan and Fang 2024; Vyas et al. 2008). Several factors may explain this difference. First, our study did not include patients younger than 6 years, in whom the cranium still predominates in size. In addition, the most severe cranial fractures may have been referred elsewhere for treatment, while the mildest cases may have gone unnoticed to avoid unnecessary radiation exposure in young children.

All in all, a study on adult facial fractures from the same region and time period than ours, found that 40% of adult subjects had mandibular fractures and 56% had midfacial fractures (Färkkilä et al. 2024). This differs from our findings, where mandibular fractures were the most common (60%) and midfacial fractures occurred in 35% of subjects. However, the divergence is coherent since, as mentioned earlier, children have smaller sinuses and a more protective cranium due to different face-to-skull bone size ratios, which prevent them from fractures in the midface (Imahara et al. 2008; Rogan et al. 2024; Rogan and Fang 2024; Totonchi et al. 2012; Vyas et al. 2008).

When examining unilateral fractures, a notable in side distribution was observed. A significant majority (80%) of fractures occurred on the left side of the head, which could be linked to the fact about 90% of people are right-handed (Levander and Schalling 1988; Papadatou-Pastou et al. 2020). Consequently, the dominant hand may instinctively protect the same side of the face in accidents, for example, directly covering the face in sports incidents or indirectly in falls or bicycle accidents. However, in MVAs for instance, the handedness does not necessarily influence trauma distribution, which may reduce the proportion of left-sided fractures.

As could be expected from the age distribution, which was weighted towards adolescents, most subjects had permanent dentition. No clear patterns between dentition stage and fracture site could be identified, partly due to the small number of subjects in most of the dentition stages. In the larger group of subjects with permanent dentition, mandibular fractures were only slightly more common than fractures in the midface or upper face, similar to the pattern observed when comparing the two age groups.

In our subjects overall, the most common trauma mechanisms were sports incidents (32%), followed by MVAs (27%) and bicycle accidents (24%). The aetiology varies somewhat between our and other´s studies. According to Irgebay et al., sports were the leading cause of injury (42%) in patients aged 12 to 18 years, whereas those younger than 6 years were mostly injured in activities of daily living (46%) (Irgebay et al. 2024). Ferreira et al. in Portugal and Grunwaldt et al. in the U. S. found MVAs to be the most common trauma mechanism (53% and 25%, respectively), whereas Ghosh et al. found falls to be the most common cause in India (59%) (Ferreira et al. 2005; Ghosh et al. 2018; Grunwaldt et al. 2011). It is also relevant to note that in our study, motorcross accidents were classified as MVAs, whereas some other studies consider them sport incidents, which affects the distribution of trauma mechanisms (Diab et al. 2021). Additionally, it is noteworthy that all our subjects who encountered assault (11%) were over 15 years old, indicating that younger patients who experienced assault did not sustain fractures.

There was a clear difference in trauma mechanisms between paediatric patients and adults from the same study period. In adults, falls (37%) and assaults (32%) were the most common causes, whereas in children, sports incidents (32%), MVAs (27%), and bicycle accidents (24%) were the leading trauma mechanisms (Färkkilä et al. 2024). This is understandable, as elderly people are more prone to get facial trauma from falls and young and middle-aged adults from assaults, whereas these causes are clearly less frequent in children.

When analysing trauma mechanisms in relation to fracture sites, bicycle accidents and assaults appeared to lead to mandibular fractures more frequently than to other facial fractures. This is supported by another study, which described the mandible as an exposed and difficult-to-protect structure (Nogami et al. 2021). Following bicycle accidents (19%), MVAs and sports related incidents (both 14%) were the next most common causes of mandibular fractures. Similarly, Smith et al. identified bicycle accidents (29%) as the most common trauma mechanism in paediatric mandibular fractures, followed by MVAs (28%) (Smith et al. 2013). In fractures of the midface or upper face, sports incidents (19%) were the leading trauma mechanism, followed by MVAs (14%).

In male subjects, the most common trauma mechanisms were sports and MVAs (both 24%) followed by bicycle accidents (16%). In contrast, in females, sports incidents and bicycle accidents were equally common (both 8.1%). This is a notable difference and can be explained by the fact that females are generally less involved in contact sports and traffic accidents than males, except in the youngest age groups (Braun et al. 2017; Grunwaldt et al. 2011). Nevertheless, it is important to keep in mind that the number of female subjects in our study is rather concise, and therefore, cannot be perfectly relied on.

Half of our subjects were treated operatively and the other half non-operatively. Only 16% of our subjects experienced AIs, suggesting that paediatric patients often confront relatively simple trauma with fewer serious AIs. The same trend applies to adults, of whom about one in five sustain AIs (Färkkilä et al. 2024). However, some studies have reported different results. For example, Grundwaldt et al. found that up to 56% of patients sustained severe concomitant injuries, most commonly soft tissue injuries and neurological trauma, with concussion being the most frequent neurological injury (Grunwaldt et al. 2011). The lower AI rate can be explained with that our AIs did not include soft tissue injuries, which are quite common in facial trauma (Mukhopadhyay et al. 2020). On the other hand, Kirvelä et al. found that 27% of paediatric patients with facial fractures had AIs and reported that the occurrence of AIs varies with age and, among teenagers, also with sex (Kirvelä et al. 2023). All our subjects had a normal GCS score of 15, which may reflect some bias, as the most severe trauma cases with lower GCSs may have been referred forward for treatment. The same applies to the fact that none of our patients sustained cervical spine injuries. In effect, this could also explain the lower AI percentage in our findings. Notably, 4 of 6 of the subjects with AIs were injured in MVAs. This is in line with other studies, as MVAs often tend to be severe, high-energy injuries (Ferreira et al. 2005). AIs most frequently affected the extremities, followed by the head and the thorax, which aligns with other studies as well (Wusiman et al. 2020).

Regarding the treatment of fractures involving the dentate regions, the major difference between distinct dentition stages is that in patients with primary or mixed dentition, titanium plates and screws must always be removed if used, to avoid interference with the developing permanent teeth. In patients with permanent dentition, the need for removal is assessed on a case-by-case basis, as the clinical situation of a 12-year-old differs from that of a 17-year-old. Open reduction and internal fixation can be more challenging in patients with mixed dentition, since screws must be placed carefully to avoid damaging permanent teeth. Additionally, fractures in paediatric patients may affect occlusion and bite development due to ongoing jaw growth.

There were notable seasonal variations in the occurrence of facial fractures. Summer (35%) and spring (30%) were the peak seasons, which can be explained by the Finnish climate that reduces outdoor activities in late autumn (22%) and especially in winter (14%). However, this should be interpreted cautiously, as some of the subjects with facial fractures during holiday periods are directly directed from PHCH to Level 1 trauma centre in Helsinki, Finland due to a lack of maxillofacial surgeon on call.

Limitations of our study include relatively small cohort size, despite a ten-year study period, as facial fractures in paediatric patients are infrequent. Another drawback is that some patients with major trauma are referred directly to tertiary hospitals. Similarly, patients with only dentoalveolar fractures are treated at various locations within the region and are therefore not certainly recorded in the PHCHs patients register. In addition, since all data was collected retrospectively, it might distort the found results slightly.

## Conclusion

Paediatric facial fractures are uncommon. In patients aged 6 to 12 years, two-thirds of the fractures involve the mandible. In the older age group, the distribution is more balanced, with mandibular fractures being slightly more frequent. No consistent association between dentition stage and fracture site is observed. In both sexes, sports related incidents are the most common trauma mechanism, followed by bicycle accidents in girls and MVAs in boys. Differences between trauma mechanisms and facial fracture locations are minor, with midfacial or upper facial fractures most often resulting from sports incidents, and mandibular fractures most commonly caused by bicycle accidents. AIs are infrequent and typically occur in connection with high-energy trauma, such as MVAs.

## Data Availability

No datasets were generated or analysed during the current study.
